# Effect of Vitamin D supplementation to reduce respiratory infections in children and adolescents in Vietnam: A randomized controlled trial

**DOI:** 10.1111/irv.12615

**Published:** 2019-01-04

**Authors:** Mark Loeb, Anh Duc Dang, Vu Dinh Thiem, Vitheya Thanabalan, Biao Wang, Nguyen Binh Nguyen, Hung Thi Mai Tran, Tan Minh Luong, Pardeep Singh, Marek Smieja, Jonathon Maguire, Eleanor Pullenayegum

**Affiliations:** ^1^ Department of Pathology and Molecular Medicine McMaster University Hamilton Ontario Canada; ^2^ National Institute of Hygiene and Epidemiology Hanoi Vietnam; ^3^ Department of Epidemiology National Institute of Hygiene and Epidemiology Hanoi Vietnam; ^4^ Department of International Cooperation National Institute of Hygiene and Epidemiology Hanoi Vietnam; ^5^ St. Michael's Hospital University of Toronto Toronto Ontario Canada; ^6^ Hospital for Sick Children University of Toronto Toronto Ontario Canada

**Keywords:** influenza, randomized trial, respiratory viruses, vitamin D

## Abstract

**Background:**

It is uncertain whether vitamin D can reduce respiratory infection.

**Objective:**

To determine whether vitamin D supplementation reduces influenza and other upper viral respiratory tract infections.

**Methods:**

A total of 1300 healthy children and adolescents between the ages of 3 and 17 years were randomized to vitamin D (14 000 U weekly) or placebo for 8 months in Vietnam. The primary outcome was reverse transcriptase (RT)‐PCR–confirmed influenza infection, and the coprimary outcome was multiplex PCR–confirmed non‐influenza respiratory viruses. Participants, caregivers, and those assessing outcomes were blinded to group assignment.

**Results:**

A total of 650 children and adolescents were randomly assigned to vitamin D and 650 to placebo. The mean baseline serum 25‐hydroxyvitamin D levels were 65.7 nmol/L and 65.2 nmol/L in the intervention and placebo groups, respectively, with an increase to 91.8 nmol/L in the vitamin D group and no increase, 64.5 nmol/L, in the placebo group. All 1300 participants randomized contributed to the analysis. We observed RT‐PCR–confirmed influenza A or B occurred in 50 children (7.7%) in the vitamin D group and in 43 (6.6%) in the placebo group (hazard ratio [HR]: 1.18, 95% CI: 0.79‐1.78). RT‐PCR–confirmed non‐influenza respiratory virus infection occurred in 146 (22.5%) in the vitamin D group and in 185 (28.5%) in the placebo group (hazard ratio [HR]: 0.76, 95% CI: 0.61‐0.94). When considering all respiratory viruses, including influenza, the effect of vitamin D in reducing infection was significant, HR: 0.81, 95% CI: 0.66‐0.99.

**Conclusion:**

Vitamin D supplementation did not reduce the incidence of influenza but moderately reduced non‐influenza respiratory viral infection.

## 1 INTRODUCTION

Influenza and other respiratory viruses account for substantial morbidity in children.^1^, ^2^, ^3^, ^4^, ^5^ The prime strategy for preventing respiratory infection is vaccination against influenza. Protection against influenza with vaccines is approximately 60% effective, but may be lower, particularly when there is a mismatch between antigens in the vaccine and the circulating influenza strains.^6^ In the absence of available vaccines against other respiratory viruses, strategies in addition to influenza vaccination may be of clinical benefit to children.

It has been proposed that vitamin D, ingested as cholecalciferol (vitamin D) or ergocalciferol (vitamin D_2_), can reduce viral respiratory infection, possibly by stimulating expression of antimicrobial peptides, such as the defensin retrocyclin‐2.^7^, ^8^ Observational studies in children have demonstrated an association between vitamin D levels and respiratory infection, but have been inconsistent.^9^, ^10^, ^11^, ^12^, ^13^, ^14^ A recent systematic review and meta‐analysis of individual participant data of vitamin D clinical trials of children and adults reported a reduced risk of acute respiratory infection (odds ratio 0.88, 95% CI: 0.81‐0.96).^15^ Important limitations were that definitions of respiratory infection in children varied considerably (eg, including pneumonia,^16^ otitis media,^17^ and exacerbation of asthma^18^, ^19^) and the vast majority of trials (23 of 25) did not include any laboratory confirmation of respiratory infection.^15^, ^20^

Conducting randomized controlled trials of vitamin D supplementation to prevent influenza and other respiratory infections can be challenging in settings where vitamin D deficiency may not be prevalent and uptake of influenza vaccination is relatively high.^21^, ^22^ In contrast, in most low‐ and middle‐income countries, such as Vietnam, children are not routinely vaccinated against influenza and vitamin D deficiency in children has been reported to be more prevalent.^23^

We conducted a randomized trial of vitamin D in children in Vietnam to assess its effectiveness in reducing laboratory‐confirmed influenza and non‐influenza viral respiratory tract infections. We hypothesized that vitamin D would reduce both laboratory‐confirmed influenza and non‐influenza respiratory viral infection compared to placebo.

## 2 METHODS

### 2.1 Study design

A placebo‐blinded randomized controlled trial. The study was conducted in two phases. In the first phase, we enrolled participants from one commune, Thanh Ha (pop. 9699), in the Thanh Liem district of Ha Nam Province. In the second phase of the trial, we enrolled participants from another commune, Kien Khe Town (pop. 9832) also in Thanh Liem district. A screening and enrollment log was used to record potential and enrolled study participants. Demographic information, past medical history, and use of prescription medications were recorded. Ethics approval was obtained at McMaster University, Hamilton, Ontario, Canada, and at National Institute of Hygiene and Epidemiology, Ministry of Health, Vietnam.

### 2.2 Participants

We enrolled children and adolescents between the ages of 3 and 17 years in Thanh Liem District of Vietnam. Thanh Liem is a rural district of Ha Nam Province, part of the Red River Delta region of Vietnam, located 50 km south of Hanoi. Children born prematurely at gestational age <32 weeks, children with any chronic illness (except asthma), children with impaired vitamin D metabolism (eg, antiseizure medications), and children with a sibling participating in the study (to reduce clustering effects) were excluded. For all participants aged 7‐17 years, an assent form was obtained. Signed parental consent was required for all participants.

### 2.3 Randomization and blinding

Participants were assigned at random to one of the two study groups (vitamin D or placebo), in a 1:1 ratio. Computer‐generated randomization was performed by an external research organization, with treatment assignments made in random permuted blocks of 4. The placebo liquid, fractionated coconut oil, was identical in appearance and taste to the vitamin D liquid. All study medication was packaged in identical‐appearing bottles. The appropriate bottle of study medication, which was stored at the commune health centers at the study sites, was labeled with the participant identification number and that individual received the contents of the pre‐labeled bottle with the participant identification. Participants were randomized in sequential order as they were enrolled. All participants and their parents, research field staff, study investigators, and research staff at McMaster University and at National Institute of Hygiene and Epidemiology were blinded.

### 2.4 Procedures

Children and adolescents randomized to vitamin D received 7 drops (0.028 mL per drop) of D drops (Ddrops Company, Woodbridge, ON) (14 000 U/wk of vitamin D) weekly for 8 months, while those randomized to placebo received 7 drops (0.028 mL per drop) of placebo drops for 8 months. The study medication was dispensed directly onto the participant's tongue. The study medication was given weekly under direct observation at the commune health center and hamlet culture houses, and missed doses were recorded.

All participants were assessed for signs and symptoms of influenza twice weekly by trained hamlet health workers over a 12‐month influenza surveillance period. This extended 4 months beyond the 8‐month period of weekly study medication. Participants were visited at home, contacted by phone, or seen at the commune health center. Each enrolled participant received a pocketed folder containing participant diary sheets, and they or their parents received instruction on how to complete these. The participant, or their parents or care providers, was responsible for completing the daily checklist which served as a memory aid. The twice‐weekly contact took place on non‐consecutive study days and optimally 3 days apart. This began after participants had been allocated to vitamin D or placebo. A standardized questionnaire was used by the trained field staff to record symptoms or signs of respiratory infection. Health workers obtained an oropharyngeal swab from participants with >1 symptoms or signs of respiratory infection including fever (temperature ≥38.0°C), cough, nasal congestion, sore throat, headache, sinus problems, muscle aches, fatigue, earache, ear infection (physician‐diagnosed), or chills. Use of an oropharyngeal swab, as opposed to nasal, was based on a high rate of positivity obtained in the pilot study with repeated specimens and perceived greater participant compliance by field staff. The specimens were stored in a refrigerator at 2‐8°C until delivery to the local laboratory which typically was within 3 hours of collection.

Serum 25(OH)D levels were assayed using the DiaSorin Vitamin D TOTAL competitive chemiluminescent immunoassay on an automated LIAISON analyzer (Stillwater, MN). A blood specimen was obtained at baseline and at 8 months after randomization. The local laboratory shipped the specimens to the laboratory at National Institute of Hygiene and Epidemiology within 24 hours of collection where the specimens were stored at ≤−70°C. These specimens were batched and then sent to McMaster University.

### 2.5 Outcomes

The primary outcome of this study was reverse transcriptase (RT)‐PCR–confirmed influenza infection using multiplex PCR for influenza A and influenza B (using modified CDC primers for matrix A gene). The coprimary outcome, non‐influenza respiratory viral infection, was measured using a separate multiplex PCR for parainfluenza 1, 2, 3, metapneumovirus, RSV, entero‐rhinovirus, and adenovirus.

Secondary outcomes included the following: influenza‐like illness, defined as cough and fever of ≥38.0°C, receipt of antibiotics, use of over the counter medication for respiratory symptoms, pharmacy visits, private medical clinic visits, and medical center or hospital visits. These outcomes were measured through parental report with confirmation through review of commune health center records when possible.

Research staff monitored for signs and symptoms of toxicity using checklists for symptoms. The major potential toxicity was hypercalcemia which could present as kidney stones. Data on toxicity were recorded monthly.

### 2.6 Statistical analysis

We calculated our sample size based on the results of the first phase of the study. We had enrolled and randomized 400 participants and followed them over 12 months. Without unblinding this first phase of the trial, we reviewed the results of samples from 260 participants who we had tested by RT‐PCR. The results showed that 81 of the 260 (31%) participants had at least one respiratory virus detected and 34 (13%) had influenza detected. We reasoned that by expanding the sample size by another 900 participants for a total of 1300 (650 participants in each group), we would have 80% power to detect a 40% risk reduction for influenza and 80% power to detect a 25% risk reduction due to vitamin D for all other respiratory viruses.

For PCR‐confirmed influenza and non‐influenza respiratory infection, we conducted a time‐to‐event analysis using Cox proportional hazards for laboratory‐confirmed influenza and for non‐influenza respiratory viruses. To avoid lack of independence associated with counting multiple outcomes, each of these outcomes in a participant was only counted once.

The secondary outcomes were treated as being dichotomous, that is, the occurrence or receipt of ≥1 of the following: influenza‐like illness, a course of antibiotics, pharmacy visit, over the counter medication for respiratory symptoms, private medical clinic visits, and hospital or medical center visits. For each of these secondary dichotomous outcomes, we estimated the absolute risk difference of the vitamin D effect.

We planned to analyze participants in the group to which they were assigned in case of crossovers. *P* values and 95% confidence intervals were calculated with 2‐tailed tests. Since the type 1 error was split between the two primary outcomes, differences with *P* < 0.025 for 2‐tailed tests were considered significant for these outcomes. For all other outcomes, differences with *P* < 0.05 were considered significant. Statistical analyses were conducted using sas version 9.2 (SAS Institute, Cary, NC) and R version 3.2.

### 2.7 Patient involvement

No patients were involved in the design of the research question, study design, or outcome measures. Because of patient preference, we did use oropharyngeal as opposed to nasopharyngeal swabs. A summary of the vitamin D levels was disseminated to participants.

## 3 RESULTS

### 3.1 Participants

**Figure 1:**
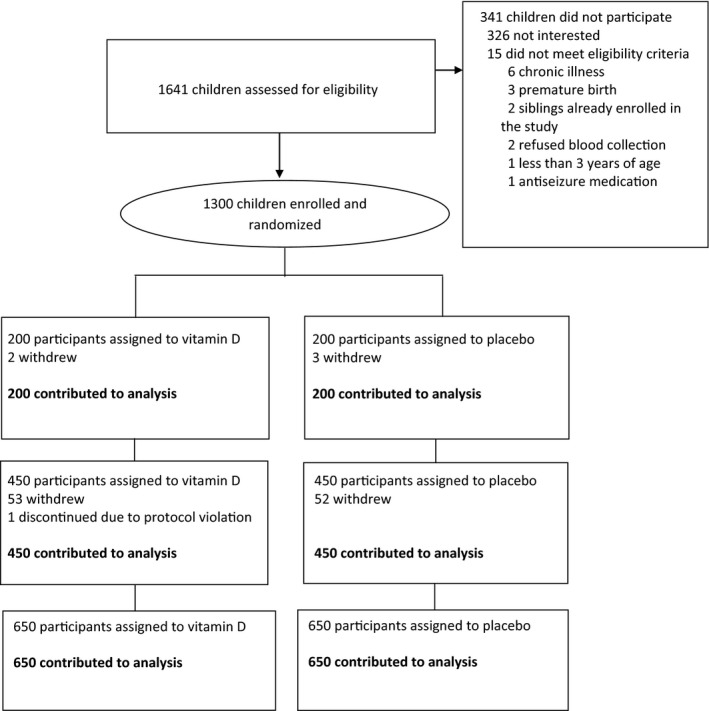
Flow of study participants: vitamin D supplementation versus placebo in children and adolescents in Vietnam

There were 1641 children and adolescents assessed for eligibility in two communes in Vietnam (Thanh Ha and Kien Khe, both in Thanh Liem district, Ha Nam province). Potentially eligible participants were pre‐screened using household demographic information from local population records. Of those assessed, 341 did not participate, 326 of whom were not interested, and 15 did not meet eligibility criteria (Figure 1). A total of 1300 were enrolled with 650 randomized to vitamin D and 650 to placebo. There were 400 participants (200 to each group) enrolled from Thanh Ha and followed from December 8, 2013, and ended on December 14, 2014. During the second year of the study, 900 new participants were enrolled from Kien Khe and randomized (450 to each group) and followed from June 2015 until June 2016 (Figure 1).

Characteristics of enrolled participants are shown in Table 1. The mean age of participants was 8.5 years (standard deviation [SD] 4.0 years); 52.2% were female; and both figures were similar between the two study groups (Table 1). Of the 1300 participants, 1073 (82.5%) had baseline and follow‐up sera for vitamin D collected. 54.8% of the participants had baseline vitamin D levels between 50 and 74 nmol/L, while 16% had levels from 25 to 49 nmol/L. Less than 1% had levels <25 nmol/L. There was an increase in the mean vitamin D level to 91.8 nmol/L (SD: 23.6 nmol/L) in the vitamin D group and no increase in the placebo group, 64.5 nmol/L (SD: 17.5 nmol/L). In the vitamin D group, 83.5% (543/650) of participants completed all of their weekly doses during follow‐up compared to 82% (533/650) in the placebo group. Of the weekly doses missed, a total of 162 were missed in 107 participants in the vitamin D group compared to 222 missed doses in 117 placebo group participants.

**Table 1:** Baseline characteristics of 1300 enrolled participants aged 3‐17 years in Vietnam

Variable	Vitamin D	Placebo
	N = 650	N = 650
Mean years of age (SD) a	8.6 (3.9)	8.4 (4.0)
3%‐6%	242 (37.2%)	245 (37.7%)
7%‐9%	171 (26.3%)	146 (22.5%)
10%‐12%	107 (16.5%)	127 (19.5%)
13%‐17%	130 (20.0%)	132 (20.3%)
Sex (Female)	325 (50.0%)	354 (54.5%)
Mean baseline vitamin D level (SD)^b^	65.73 (16.72)	65.21 (16.89)
<25	3 (0.54%)	3 (0.55%)
25‐49	92 (16.64%)	86 (15.87%)
50‐74	300 (54.25%)	296 (54.61%)
≥75	158 (28.57%)	157 (28.97%)

a Standard deviation.

b Based on 553/650 (85.1%) samples from intervention group and 542/650 (83.4%) in the placebo group.

Mean (SD) duration of follow‐up was similar between groups: 331.9 (87.3) days in the vitamin D group and 332.9 (85.1) days in the placebo group. Of enrolled participants, 1189 (91.5%) completed follow‐up over the 12‐month period. Of the 111 participants who withdrew from the study, 56 were in the vitamin D group and 55 in the placebo group. None of these withdrawals were due to adverse events. Reasons for withdrawal are shown in Figure 1. There were 1697 oropharyngeal specimens obtained (854 in the vitamin D group and 843 in the placebo group).

### 3.2 Outcomes

We observed RT‐PCR–confirmed influenza A or B in 50 children (7.7%) in the vitamin D group and in 43 children (6.6%) in the placebo group (Table 2). Of these, 67 (72.0%) had influenza A, 24 (28.8%) had influenza B, and 2 (2.2%) had both influenza A and B. Non‐influenza respiratory virus infection occurred in 146 (22.5%) in the vitamin D group and in 185 (28.5%) in the placebo group (Table 3). There were a total of 177 (27.2%) influenza and non‐influenza respiratory viruses in the vitamin D group and 209 (32.2%) in the placebo group.

**Table 2:** Effectiveness of vitamin D supplementation on reducing RT‐PCR–confirmed influenza and non‐influenza respiratory infections

Outcome	RT‐PCR–confirmed infection		HR^a^ (95% CI)
	Vitamin D	Placebo	
Non‐influenza virus infection			
All Years	146/650 (22.5%)	185/650 (28.5%)	0.76 (0.61‐0.94)
Year 1	54/200 (27.0%)	75/200 (37.50%)	0.65 (0.46‐0.93)
Year 2	92/450 (20.4%)	110/450 (24.4%)	0.82 (0.62‐1.08)
Influenza virus infection			
All Years	50/650 (7.7%)	43/650 (6.6%)	1.18 (0.79‐1.77)
Year 1	25/200 (12.5%)	29/200 (14.5%)	0.85 (0.50‐1.46)
Year 2	25/450 (5.6%)	14/450 (3.1%)	1.82 (0.95‐3.52)
All viral infections			
All Years	177/650 (27.2%)	209/650 (32.2%)	0.81 (0.67‐0.99)
Year 1	72/200 (36.0%)	90/200 (45.0%)	0.73 (0.53‐0.99)
Year 2	105/450 (23.3%)	119/450 (26.4%)	0.87 (0.67‐1.13)

a All hazard ratios were calculated using the participants’ first infection with the virus.

**Table 3:** The distribution of RT‐PCR–confirmed respiratory viral infection in participants by vitamin D group and placebo group^a^

Variable	Vitamin D	Placebo
	N = 650	N = 650
≥1 virus	177 (27.2%)	209 (32.2%)
Influenza A or B	50 (7.7%)	43 (6.6%)
Influenza A	40 (6.2%)	29 (4.5%)
Influenza B	11 (1.7%)	15 (2.3%)
Adenovirus	10 (1.5%)	11 (1.7%)
Entero‐rhino	130 (20.0%)	159 (24.5%)
MPV	12 (1.8%)	25 (3.8%)
Parainfluenza 1	1 (0.2%)	0 (0.0%)
Parainfluenza 2	0 (0.0%)	2 (0.3%)
Parainfluenza 3	12 (1.8%)	9 (1.4%)
RSV	7 (1.1%)	10 (1.5%)

a The sum of participants with confirmed infection with the different types of viruses is greater than the number for ≥1 virus because of those participants infected with more than one type of virus.

**Figure 2:**
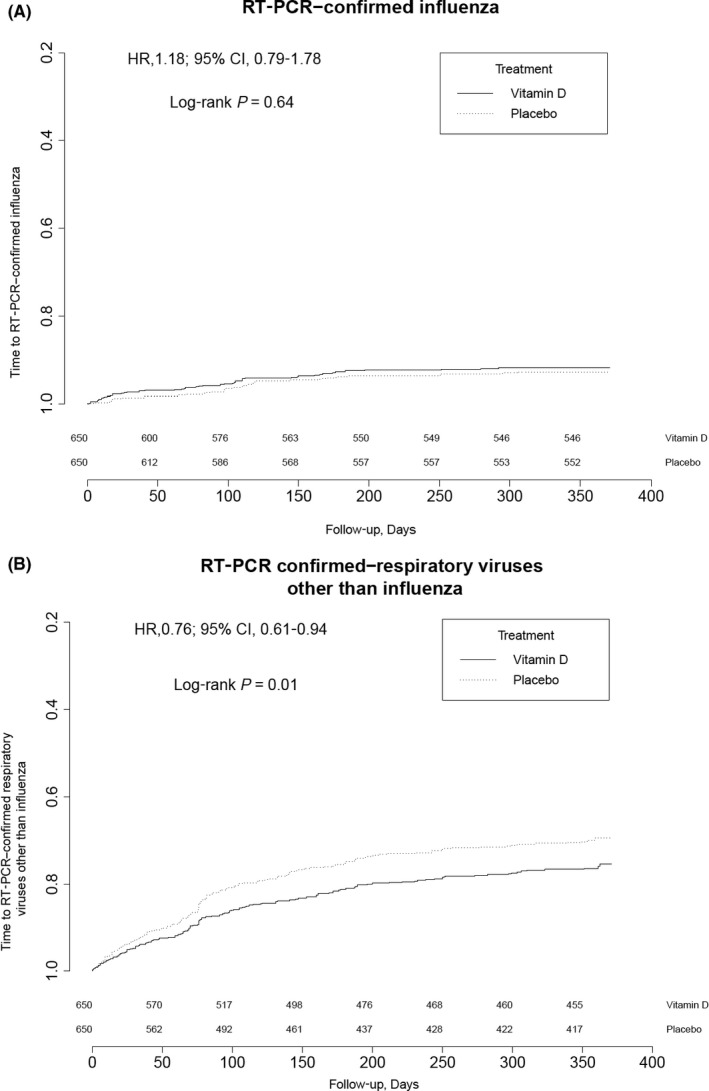
Kaplan‐Meier curve of time to first RT‐PCR–confirmed A. influenza A or B infection and B, non‐influenza respiratory viruses

We found no significant difference between vitamin D and placebo groups for RT‐PCR–confirmed influenza, hazard ratio [HR]: 1.18, 95% CI: 0.79‐1.77 (Figure 2). We found that vitamin D significantly reduced non‐influenza respiratory viral infection, HR: 0.76, 95% CI: 0.61‐0.94 (*P* = 0.011). Although the attack rates differed between the two stages of the study, the relationship between the two groups was similar (Table 2). When considering all respiratory viruses, including influenza, the effect of vitamin D in reducing infection was significant, HR: 0.81, 95% CI: 0.66‐0.99.

When we compared the vitamin D group to the placebo group for secondary outcomes, we found the following: 50 (7.7%) vs 62 (9.5%) with ≥ 1 episodes of influenza‐like illness (absolute difference −1.8%, 95% CI: −4.9%‐1.2%), 270 (41.5%) vs 282 (43.4%) who received ≥ 1 course of antibiotics (absolute difference −1.85%, 95% CI: −7.2%‐3.5%), 245 (37.7%) vs 251 (38.6%) that visited a pharmacy because of respiratory infection (absolute difference −0.92%, 95% CI: −6.2%‐4.4%), and 338 (52.0%) vs 361 (55.5%) that used over the counter medications for respiratory infection (absolute difference −3.5%, 95% CI: −9.0%‐1.9%). No significant difference in these outcomes between groups was found. Only two participants in each group visited a private clinic, and only one in the vitamin D group and two in the placebo visited a hospital or medical center for respiratory symptoms. Only one serious adverse event was reported, which was not related to the study, a hospitalization for a scheduled tonsillectomy.

We conducted a post hoc subgroup analysis limited to participants who at baseline had 25OHD levels less than 50 nmol/L. In this cohort, there were only 14 cases of influenza, and the hazard ratio was 2.5 (5% CI: 0.78‐7.9), *P* = 0.12. When we conducted the analysis using other respiratory viruses as the outcome, there were 46 events, HR: 0.92, 95% CI: 0.52‐1.66, *P* = 0.8.

## 4 DISCUSSION

We found that vitamin D supplementation of 14 000 U/wk for 8 months had no significant effect on confirmed influenza infection in healthy children and adolescents between the ages of 3 and 17 years in Vietnam. However, supplementation significantly reduced RT‐PCR–confirmed non‐influenza respiratory viral infections by about 25%.

### 4.1 Strengths and limitations of the study

Strengths of the study were that it was a randomized placebo‐controlled trial with laboratory‐confirmed respiratory viral infection as the main outcome and measurement of vitamin both at baseline and at follow‐up.

One possible limitation was the use of oropharyngeal specimens instead of nasopharyngeal specimens. This was based on adherence to testing, and we reasoned that an increase in the number of swabs would make up for any reduction in sensitivity. The attack rate for all respiratory viruses in our study was 35%, which is comparable to other studies that have used RT‐PCR to detect infection in children.^24^, ^25^, ^26^ A study in the same population in Vietnam reported similar attack rates as ours.^23^ Also, detection of the types of viruses and their relative frequency was similar to previous reports. The most common non‐influenza respiratory virus we detected was entero‐rhinovirus, consistent with other studies.^24^, ^25^, ^26^ In our study, the ratio of attack rates for vitamin D to placebo for entero‐rhinovirus, 0.83, represented the median effect for non‐influenza respiratory viruses, which ranged from 0.47 for human metapneumovirus to 1.17 for parainfluenza viruses 1‐3.

Only 17% of participants in our study had vitamin D levels <50 nmol/L, in contrast to a previous study from Vietnam where >50% children had insufficiency or deficiency.^23^ The relatively high vitamin D levels in our study may have reduced the effectiveness of vitamin D supplementation in preventing influenza. In contrast, vitamin D supplementation demonstrated effectiveness in reducing non‐influenza respiratory viral infection. Although vitamin D may have variable immunomodulatory effect on different respiratory viruses,^27^ we are unaware of a specific mechanism that would explain the differential effect we observed. The lower confidence interval for the effect of vitamin D on influenza, 0.79, along with the point estimate of 1.18, suggests that these data are unlikely to be compatible with a large protective effect of influenza.

### 4.2 Comparison with other studies

Two previous trials of vitamin D in children reported either an effect of vitamin D on all respiratory viruses or no effect on all influenza.^28^, ^29^ These studies had limitations such as lack of RT‐PCR testing,^28^, ^29^ survey‐reported symptom outcomes at the end of the study,^28^ and the lack of criteria for ascertaining outcomes^29^ that limit inferences and comparisons to our trial. A randomized trial of high‐dose versus standard‐dose vitamin D supplementation in Canadian young children did not demonstrate a reduction of overall wintertime upper respiratory tract infections.^30^ Our study was conducted in a middle‐income country where children and adolescents are not routinely vaccinated against influenza. Vitamin D levels, while not as low as anticipated, may still be lower in this population than in many settings in developed countries. Therefore, our results may apply to children and adolescents in other low‐ and middle‐income countries.

## 5 CONCLUSION

Our results show that vitamin D supplementation does not reduce influenza but can reduce non‐influenza respiratory infections in children and adolescents aged 3‐17 years in a low‐ and middle‐income country. Our findings imply that vitamin D supplementation can play a moderate role in reducing illness caused by respiratory viruses.

### CONFLICT OF INTEREST

All authors have completed the ICMJE uniform disclosure form at www.icmje.org/coi_disclosure.pdf and declare: All authors had support from Infectious Diseases Research at McMaster University that funded the research and the Ddrops Company that provided the vitamin D for the submitted work; no financial relationships with any organizations that might have an interest in the submitted work in the previous 3 years; no other relationships or activities that could appear to have influenced the submitted work.

## AUTHOR CONTRIBUTIONS

ML, DDA, MS, and JM conceived and designed the study. VDT, VT, NBN, TTMH, LMT, and SE were responsible for acquisition of data. ML, BW, PS, and EP were responsible for the analysis and interpretation of data. ML produced a first draft of the manuscript, and all authors provided intellectual input. ML is the guarantor.

## COPYRIGHT

The Corresponding Author has the right to grant on behalf of all authors and does grant on behalf of all authors, *a worldwide licence* to the Publishers and its licensees in perpetuity, in all forms, formats, and media (whether known now or created in the future), to (a) publish, reproduce, distribute, display, and store the Contribution; (b) translate the Contribution into other languages, create adaptations, reprints, include within collections, and create summaries, extracts, and/or abstracts of the Contribution; (c) create any other derivative work(s) based on the Contribution; (d) exploit all subsidiary rights in the Contribution; (e) the inclusion of electronic links from the Contribution to third party material wherever it may be located; and (f) license any third party to do any or all of the above.

## ETHICAL APPROVAL

The study was approved by the research ethics board of McMaster University and that of the Ministry of Health in Vietnam.

## DATA SHARING

Patient‐level data and statistical code are available upon request from the corresponding author at loebm@mcmaster.ca.

## TRANSPARENCY

Lead author (ML) affirms that the manuscript is an honest, accurate, and transparent account of the study being reported; that no important aspects of the study have been omitted; and that any discrepancies from the study as planned (and, if relevant, registered) have been explained.

## References

[1] Lafond KE , Nair H , Rasooly MH , et al. Global role and burden of influenza in pediatric respiratory hospitalizations, 1982‐2012: a systematic analysis. PLoS Med. 2016;13(3):e1001977. 27011229 10.1371/journal.pmed.1001977 PMC4807087

[2] Shi T , Balsells E , Wastnedge E , et al. Risk factors for respiratory syncytial virus associated with acute lower respiratory infection in children under five years: systematic review and meta‐analysis. J Glob Health. 2015;5(2):020416. 26682048 10.7189/jogh.05.020416 PMC4676580

[3] de Francisco Shapovalova SN , Donadel M , Jit M , Hutubessy R . A systematic review of the social and economic burden of influenza in low‐ and middle‐income countries. Vaccine. 2015;33(48):6537‐6544. 26597032 10.1016/j.vaccine.2015.10.066

[4] Thompson WW , Shay DK , Weintraub E , et al. Influenza‐associated hospitalizations in the United States. JAMA. 2004;292:1333‐1340. 15367555 10.1001/jama.292.11.1333

[5] Izurieta HS , Thompson WW , Kramarz P , et al. Influenza and the rates of hospitalization for respiratory disease among infants and young children. N Engl J Med. 2000;342:232‐239. 10648764 10.1056/NEJM200001273420402

[6] Osterholm MT , Kelley NS , Sommer A , Belongia EA . Efficacy and effectiveness of influenza vaccines: a systematic review and meta‐analysis. Lancet Infect Dis. 2012;12(1):36‐44. 22032844 10.1016/S1473-3099(11)70295-X

[7] Sundaram ME , Coleman LA . Vitamin D and influenza. Adv Nutr. 2012;3(4):517‐525. 22797987 10.3945/an.112.002162 PMC3649720

[8] Khoo AL , Chai L , Koenen H , Joosten I , Netea M , van der Ven A . Translating the role of vitamin D3 in infectious diseases. Crit Rev Microbiol. 2012;38:122‐135. 22304022 10.3109/1040841X.2011.622716

[9] Wayse V , Yousafzai A , Mogale K , Filteau S . Association of subclinical vitamin D deficiency with severe acute lower respiratory infection in Indian children under 5 y. Eur J Clin Nutr. 2004;58:563‐567. 15042122 10.1038/sj.ejcn.1601845

[10] Karatekin G , Kaya A , Salihoğlu O , Balci H , Nuhoğlu A . Association of subclinical vitamin D deficiency in newborns with acute lower respiratory infection and their mothers. Eur J Clin Nutr. 2009;63:473‐477. 18030309 10.1038/sj.ejcn.1602960

[11] McNally JD , Leis K , Matheson LA , Karuananyake C , Sankaran K , Rosenberg AM . Vitamin D deficiency in young children with severe acute lower respiratory infection. Pediatr Pulmonol. 2009;44:981‐988. 19746437 10.1002/ppul.21089

[12] Roth DE , Shah R , Black RE , Baqui AH . Vitamin D status and acute lower respiratory infection in early childhood in Sylhet, Bangladesh. Acta Paediatr. 2010;99:389‐393. 19900174 10.1111/j.1651-2227.2009.01594.x

[13] Roth DE , Jones AB , Prosser C , Robinson JL , Vohra S . Vitamin D status is not associated with the risk of hospitalization for acute bronchiolitis in early childhood. Eur J Clin Nutr. 2009;63(2):297‐299. 17971825 10.1038/sj.ejcn.1602946

[14] Science M , Maguire JL , Russell ML , Smieja M , Walter SD , Loeb M . Low serum 25‐hydroxyvitamin D level and risk of upper respiratory tract infection in children and adolescents. Clin Infect Dis. 2013;5:392‐397. 10.1093/cid/cit289 PMC3888147 23677871

[15] Martineau AR , Jolliffe DA , Hooper RL , et al. Vitamin D supplementation to prevent acute respiratory tract infections: systematic review and meta‐analysis of individual participant data. BMJ. 2017;356:i6583. 28202713 10.1136/bmj.i6583 PMC5310969

[16] Manaseki‐Holland S , Maroof Z , Bruce J , et al. Effect on the incidence of pneumonia of vitamin D supplementation by quarterly bolus dose to infants in Kabul: a randomised controlled superiority trial. Lancet. 2012;356:1419‐1427. 10.1016/S0140-6736(11)61650-4 PMC3348565 22494826

[17] Marchisio P , Consonni D , Baggi E , et al. Vitamin D supplementation reduces the risk of acute otitis media in otitis‐prone children. Pediatr Infect Dis J. 2013;356:1055‐1060. 10.1097/INF.0b013e31829be0b0 23694840

[18] Majak P , Olszowiec‐Chlebna M , Smejda K , Stelmach I . Vitamin D supplementation in children may prevent asthma exacerbation triggered by acute respiratory infection. J Allergy Clin Immunol. 2011;356:1294‐1296. 10.1016/j.jaci.2010.12.016 21315433

[19] Tachimoto H , Mezawa H , Segawa T , Akiyama N , Ida H , Urashima M . Improved control of childhood asthma with low‐dose, short‐term vitamin D supplementation: a randomized, double‐blind, placebo‐controlled trial. Allergy. 2016;356:1001‐1009. 10.1111/all.12856 26841365

[20] Bolland MJ , Avenell A . Do vitamin D supplements help prevent respiratory infections? BMJ. 2017;356:j456. 28202434 10.1136/bmj.j456

[21] Kumar J , Muntner P , Kaskel FJ , Hailpern SM , Melamed ML . Prevalence and associations of 25‐hydroxyvitamin D deficiency in US children: NHANES 2001‐2004. Pediatrics. 2009;124:e362‐e370. 19661054 10.1542/peds.2009-0051 PMC3749840

[22] CDC Flu vaccination coverage, United States, 2015‐2016 influenza season. http://www.cdc.gov/flu/fluvaxview/coverage-1516estimates.htm. Accessed October 22, 2018.

[23] Laillou A , Wieringa F , Tran TN , et al. Hypovitaminosis D and mild hypocalcaemia are highly prevalent among young Vietnamese children and women and related to low dietary intake. PLoS One. 2013;8(5):e63979. 23717521 10.1371/journal.pone.0063979 PMC3663760

[24] Monto AS , Malosh RE , Petrie JG , Thompson MG , Ohmit SE . Frequency of acute respiratory illnesses and circulation of respiratory viruses in households with children over 3 surveillance seasons. J Infect Dis. 2014;210:1792‐1799. 24907381 10.1093/infdis/jiu327 PMC4296188

[25] Do AH , van Doorn HR , Nghiem MN , et al. Viral etiologies of acute respiratory infections among hospitalized Vietnamese children in Ho Chi Minh City, 2004–2008. PLoS One. 2011;6:e18176. 21455313 10.1371/journal.pone.0018176 PMC3063798

[26] Nguyen DNT , Mai LQ , Bryant JE , et al. Epidemiology and etiology of influenza‐like‐illness in households in Vietnam; it's not all about the kids!. J Clin Virol. 2016;82:126‐132. 27479176 10.1016/j.jcv.2016.07.014 PMC4994428

[27] Greiller CL , Martineau AR . Modulation of the immune response to respiratory viruses by vitamin D. Nutrients. 2015;7:4240‐4270. 10.3390/nu7064240 26035247 PMC4488782

[28] Camargo CA Jr , Ganmaa D , Frazier AL , et al. Randomized trial of vitamin D supplementation and risk of acute respiratory infection in Mongolia. Pediatrics. 2012;130(3):e561‐e567. 22908115 10.1542/peds.2011-3029

[29] Urashima M , Segawa T , Okazaki M , Kurihara M , Wada Y , Id H . Randomized trial of vitamin D supplementation to prevent seasonal influenza A in schoolchildren. Am J Clin Nutr. 2010;91:1255‐1260. 20219962 10.3945/ajcn.2009.29094

[30] Agilpay M , Birken CS , Parkin PC , et al. Effect of High‐Dose vs Standard‐Dose Wintertime Vitamin D Supplementation on Viral Upper Respiratory Tract Infections in Young Healthy Children. JAMA. 2017;318:245‐254. 28719693 10.1001/jama.2017.8708 PMC5817430

